# Molecularly Imprinted Polymer Sensor Empowered by Bound States in the Continuum for Selective Trace‐Detection of TGF‐beta

**DOI:** 10.1002/advs.202401843

**Published:** 2024-09-05

**Authors:** Gianluigi Zito, Giulia Siciliano, Aida Seifalinezhad, Bruno Miranda, Vittorino Lanzio, Adam Schwartzberg, Giuseppe Gigli, Antonio Turco, Ivo Rendina, Vito Mocella, Elisabetta Primiceri, Silvia Romano

**Affiliations:** ^1^ Institute of Applied Sciences and Intelligent Systems National Research Council Via Pietro Castellino 111 Napoli 80131 Italy; ^2^ Institute of Nanotechnology National Research Council c/o Campus Ecotekne, Via Monteroni Lecce 73100 Italy; ^3^ Department of Engineering Università degli Studi di Napoli Parthenope Centro Direzionale di Napoli, Isola C4 Naples 80143 Italy; ^4^ Molecular Foundry Lawrence Berkeley National Laboratory 1 Cyclotron Rd Berkeley CA 94720 USA

**Keywords:** biosensing, bound states in the continuum, cytokine, molecularly imprinted polymer

## Abstract

The integration of advanced materials and photonic nanostructures can lead to enhanced biodetection capabilities, crucial in clinical scenarios and point‐of‐care diagnostics, where simplified strategies are essential. Herein, a molecularly imprinted polymer (MIP) photonic nanostructure is demonstrated, which selectively binding to transforming growth factor‐beta (TGF‐β), in which the sensing transduction is enhanced by bound states in the continuum (BICs). The MIP operating as a synthetic antibody matrix and coupled with BIC resonance, enhances the optical response to TGF‐β at imprinted sites, leading to an augmented detection capability, thoroughly evaluated through spectral shift and optical lever analogue readout. The validation underscores the MIP‐BIC sensor capability to detect TGF‐β in spiked saliva, achieving a limit of detection of 10 fM and a resolution of 0.5 pM at physiological concentrations, with a precision of two orders of magnitude above discrimination threshold in patients. The MIP tailored selectivity is highlighted by an imprinting factor of 52, showcasing the sensor resistance to interference from other analytes. The MIP‐BIC sensor architecture streamlines the detection process eliminating the need for complex sandwich immunoassays and demonstrates the potential for high‐precision quantification. This positions the system as a robust tool for biomarker detection, especially in real‐world diagnostic scenarios.

## Graphical abstract

Combining molecular imprinting polymers and refractometric optical sensing based on bound states in the continuum allows demonstrating a new platform for ultra‐sensitive biodetection, which is capable of large selectivity and specificity necessary in clinical scenarios. The device is applied for detecting TGF‐beta cytokine in saliva outperforming state‐of‐the‐art technologies.

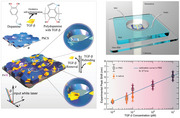

## 1 Introduction

In the quest for highly sensitive, label‐free refractometric sensors, the integration of advanced materials and innovative optical structures has become imperative to push the limits of detection in challenging applications, such as cancer biomarker sensing. Oral cancer is one of the most common malignancies worldwide, with 300,000 diagnoses and 145,000 deaths per year.^[^ ^1^ ^]^ Cytokines, such as transforming growth factor‐beta (TGF‐β), are signaling molecules produced by various cells in the immune system and other tissues. TGF‐β in particular is involved in the formation of the extracellular matrix and can facilitate tumor progression by promoting the epithelial‐mesenchymal transition, which enhances cancer cell invasion and metastasis.^[^ ^2^ ^]^ Saliva is an attractive source for biomarker discovery and detection in oral cancer due to its accessibility and non‐invasive collection method. TGF‐β and other cytokines can be measured in saliva samples, and their levels may provide valuable information for oral cancer diagnosis,^[^ ^3^ ^]^ prognosis, and disease monitoring.^[^ ^4^ ^]^ However, the concentration of TGF‐β in the saliva is in the range of nanograms per milliliter (picomolar range).^[^ ^5^ ^]^ To attain precise quantification with elevated specificity, there is a pressing need to develop novel diagnostic tools, designed for streamlined and scalable point‐of‐care diagnostics, ensuring their efficacy in diverse healthcare settings.

This paper endeavors to trace a transformative sensing strategy by integrating molecularly imprinted polymers (MIPs) and photonic bound states in the continuum (BICs).

Although embedded in the continuum of radiative waves, photonic BICs are ideal dark modes that remain confined with infinite radiative *Q*_*r*_‐factor.^[^ ^6^, ^7^, ^8^, ^9^, ^10^, ^11^, ^12^, ^13^ ^]^ In particular, these special ultranarrow optical resonances are associated with nontrivial topological properties.^[^ ^14^, ^15^, ^16^, ^17^ ^]^ They offer the possibility of creating novel photonic systems by effectively trapping optical energy and increasing light‐matter interactions,^[^ ^18^, ^19^, ^20^, ^21^, ^22^ ^]^ thus enhancing the efficiency in linear and nonlinear optical phenomena.^[^ ^23^, ^24^, ^25^, ^26^ ^]^ Furthermore, dielectric resonant nanostructures that sustain BICs have emerged as a highly sensitive technology for in situ measurement of surface material interactions.^[^ ^27^, ^28^, ^29^, ^30^, ^31^, ^32^, ^33^, ^34^, ^35^, ^36^ ^]^ Indeed, the high radiative *Q*_*r*_‐factor arising from the decoupling with far‐field radiation because of symmetry protection^[^ ^9^ ^]^ or interference mechanisms^[^ ^26^ ^]^ in real systems sustaining quasi‐BICs, allows to establish highly‐sensitive detection strategies with large‐area and facile interrogation schemes.^[^ ^37^ ^]^ Noteworthy attributes of BIC‐based sensors include label‐free, loss‐free, and real‐time measurements, making them powerful tools for sensing biomolecular interactions in medicine^[^ ^37^, ^38^ ^]^ and applications related to food control.^[^ ^39^ ^]^

MIPs are synthetic receptors utilized as mimetic antibodies for selective molecular recognition, embedding sites complementary to the target molecule for size, shape, and functionality.^[^ ^40^, ^41^ ^]^ Several works have reported the use of MIPs based on dopamine (DA) self‐polymerization for detecting various target molecules, such as 17β‐oestradiol,^[^ ^42^ ^]^ bovine serum albumin,^[^ ^43^ ^]^ C‐reactive protein,^[^ ^44^ ^]^ and sulfamethoxazole.^[^ ^45^ ^]^ In addition, DA is a highly biocompatible and water‐soluble functional monomer capable of self‐polymerizing on different substrates in mild conditions. These properties have made DA a suitable matrix for the preparation of protein‐selective MIPs used for biosensing applications^[^ ^46^, ^47^ ^]^ and innovative transducers, which are widely sought after to improve the capability of analyte recognition. Indeed, the utilization of MIPs offers remarkable selectivity and affinity, making them ideal candidates for specific target detection. In addition, the long‐term storage stability, tailoring flexibility, low‐cost production, and potential reusability of MIPs are important factors to be accounted for in several sensing applications,^[^ ^48^, ^49^ ^]^ drug delivery systems,^[^ ^50^, ^51^ ^]^ and analysis of protein biomarkers.^[^ ^52^ ^]^

Herein, we develop an all‐dielectric nanostructure sensor based on BICs and based on a photonic crystal slab (PhCS) thoroughly engineered with a imprinting layer of polydopamine (PDA) for specific binding to TGF‐β. The polydopamine MIP plays a dual role in this system. Functioning as the molecular recognition element, it selectively captures the analyte, and precisely where the optical resonant field is most intense. The BIC, with its local field enhancement and confined light, intensifies the interaction with the imprinted sites on the polymer, significantly amplifying the signal associated with TGF‐β binding. Thus, MIP increases the sensitivity of the BIC‐based sensor to minute refractive index variations associated with TGF‐β binding with no need for long and complex functionalization steps while ensuring high specificity with negligible interference from other analytes. To elucidate the performance of the proposed device, we investigate its limit of detection (LOD) for TGF‐β. The LOD is carefully characterized, considering factors such as signal‐to‐noise ratio, response time, reproducibility, and differential readout analyses including spectral shift and optical lever analog, providing a comprehensive assessment of the analytical capabilities. Finally, we demonstrate the TGF‐β detection in a complex matrix of spiked saliva with a LOD = 10 fM with the spectral shift readout. Also, a high resolution (0.5 pM) at physiological concentration is shown using the optical lever readout, which is associated to the sensorcapability of precised assessment of TGF‐β levels. Such a high resolution is necessary to accurately discriminate normal from abnormal levels in patients. In addition, it is worth stressing that the lowest LOD of 20 fM in saliva has been reported by Sanchez et al. using an electrochemical immunosensor based on a sandwich immunoassay with carbon nanotubes conjugated with TGF‐β antibody as carrier tags.^[^ ^53^ ^]^ Therefore, our MIP‐BIC sensor outperforms the state of the art and introduces a fundamental simplification using a sensing architecture that does not necessitate a sandwich immunoassay. The femtomolar sensitivity makes it a powerful tool for biomarker detection in various clinical scenarios, offering the needed precision for valid label‐free quantification.

## 2 Results and Discussion

### 2.1 Rationale

**Figure 1:**
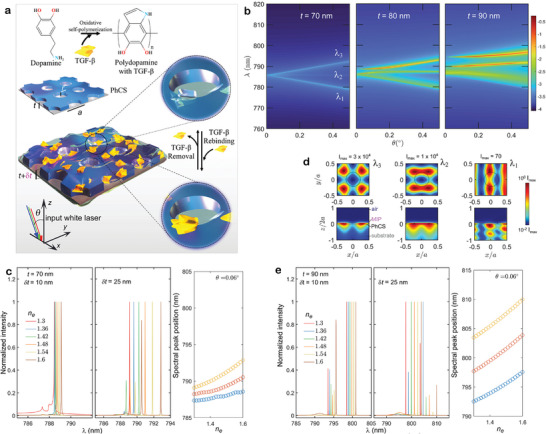
a) Scheme of MIP‐BIC sensor for TGF‐β detection, the incidence angle θ lies in the *xz*‐plane. b) Evolution of the principal dispersion absorptance curves log_10_ *A*(θ) (colorbar log scale) with the thickness of the slab *t*. c) Simulated spectra for δ*t* = 10 nm and 25 nm, with correspondent peak evolution as a function of the cladding effective refractive index *n*_*e*_ for the three modes λ_1_ (cyan), λ_2_ (red), λ_3_ (orange), estimated considering an incident angle close to the normal (θ = 0.06°). d) Near field intensity cross sections for the modes λ_1_, λ_2_, and λ_3_ simulated for *t* = 90 nm (experimental case) at the *xy*‐plane: *z*/*a* = 0, and *xz*‐plane: *y*/*a* = 0.25. (e) Same as in (c) for *t* = 90 nm, corresponding to the experimental structure actually used.

The sensing transduction mechanism of a spectral refractometric sensor relies on the spectral shift variation of the resonant wavelength of the sensor in response to variations in the target analyte concentration. A narrow resonance allows quantifying the analyte concentration accurately.^[^ ^38^, ^39^ ^]^ A larger spectral shift corresponds to a higher concentration of the target analyte. The larger the spectral shift for fixed concentration, the larger the sensorsensitivity. **Figure** **1**a illustrates the selective binding principle of TGF‐β through the MIP. The PhCS design followed a specific strategy. Initially, resonances were designed in the near‐infrared spectral range, exploiting the sensitivity increase with the wavelength (λ).^[^ ^29^ ^]^ For a sufficiently small thickness of the patterned silicon nitride slab (i.e., *t* = 70 nm when *a* = 540 nm), the three principal dispersion curves are degenerate at the **Γ** point in momentum space, supporting the formation of a Dirac cone.^[^ ^37^ ^]^ In Figure 1b, the evolution of the bands with increasing slab thickness *t* is presented. Instead of typical reflectance spectra *R*_*s*_ (s‐polarization, TE‐like modes), we depict absorptance in logarithmic scale, log_10_[*A*(λ)] = log_10_[1 − *T*_*s*_(λ) − *R*_*s*_(λ)] (with *T*_*s*_ as the associated transmittance). This representation better reflects the real position of the maximum field of generally asymmetric Fano‐shaped resonances and simultaneously provides an estimate of the local field intensity (proportional to *A*(λ)), crucial for enhancing sensing capabilities.

As evidenced from Figure 1c, even under significant perturbation of the refractive index of the MIP imprinting layer, the Dirac cone degeneracy remains stable as the spectral position of the modes is minimally affected (left inset). Only with a larger imprinting thickness of δ*t* = 25 nm does significant spectral mode splitting occur (right inset). Deliberately breaking the Dirac cone with increasing *t* results in two emerging BICs at λ_2_ and λ_3_, situated at lower energies than the dipole lossy band at λ_1_ (Figure 1d,e). This yields two resonant peaks with an ultra‐high *Q*_*r*_‐factor. The mechanism is further detailed in the Experimental Section. While the position in the energy‐momentum space of the field intensity maximum may vary with cladding parameters (and hence the concentration of target molecules and geometry of the MIP), its value consistently exceeds the dipolar mode by three orders of magnitude. In addition, its spectral position always falls within 0.5 degrees from **Γ** point (incidence angle θ = 0). In particular, in Figure 1d, the maximum occurs at θ^⋆^ = 0.031° with no MIP (δ*t* = 0). Generally, the maximum intensity is reached at the critical coupling condition where the radiative quality factor matches the nonradiative one (*Q*_*r*_ = *Q*_*a*_).^[^ ^33^, ^34^ ^]^ Since *Q*_*r*_ varies as a power of the inverse momentum displacement from the BIC‐momentum point,^[^ ^24^ ^]^ in lossy systems, the maximum field is typically achieved only far from the high‐symmetry point of symmetry‐protected BICs or through in‐plane inversion symmetry‐breaking with asymmetric unit cells.^[^ ^54^ ^]^ Our case is distinctive. Indeed, accurate fabrication of the silicon nitride ensures that the imaginary part of the measured slab refractive index (representing nonradiative quality factor *Q*_*a*_) remains below 10^−6^ in the near‐infrared (Experimental Section). This unique feature allows radiative and nonradiative losses to intersect near the symmetry point (θ^⋆^ = 0.031°), eliminating the need for complex symmetry breaking.

The theoretical investigation into the BIC sensor dependence on the refractive index of the MIP layer proceeded as outlined below. The refractive index of the MIP under conditions of high density and uniformity was estimated at *n*_MIP_ = 1.74.^[^ ^55^ ^]^ Employing a Langmuir isotherm to model the surface binding process, which is proportional to the concentration *C* of the analyte molecules in the solvent, the binding of the analyte molecules was represented through an effective refractive index *n*_*e*_(*C*). This factor accounts for the conformal layer of the MIP and MIP voids created by the template molecules subsequently filled with TGF‐β during resorption. The asymptotic adsorption level corresponded to the MIP saturated with molecules, forming a monolayer. An upper limit for the filling fraction of TGF‐β in the MIP was set at 60% due to random close packing. Consequently, the resorption of TGF‐β into the MIP was simulated by adjusting the effective refractive index of the cladding, with *n*_*e*_ ranging from 1.3 (corresponding to a MIP matrix with 60% voids) to 1.6, representing a MIP matrix with 60% filled with TGF‐β having a refractive index of *n*_tgf_ = 1.5. Details of this simulation can be found in the Supporting Information, and Figure S1 (Supporting Information) illustrates the variation of *n*_*e*_(*C*) with TGF‐β concentration.

Numerical simulations were carried out to investigate the shift of resonant peaks concerning both the MIP thickness (δ*t*) and the effective refractive index of the imprinting layer. As shown in Figure S2 (Supporting Information), an impressive shift of 25 nm could be achieved with an MIP thickness on the order of 100 nm upon complete TGF‐β adsorption. However, considering the molecular weight of 44 kDa, the estimated volume and coverage area of TGF‐β were 53 nm^3^ and 17 nm^2^, respectively. Consequently, our experimental focus was directed toward a sufficiently low MIP thickness to effectively capture the target molecules in close proximity to the PhCS. The experimentally measured MIP thickness ranged from 10 to 25 nm, fitting better with the size of the target molecule and facilitating its steric inclusion. Considering δ*t* = 25 nm, the resulting modulation of the peak position is expected to span over 6.5 nm (Figure 1e; Figure S2, Supporting Information). Figure 1e provides detailed insight into the associated exponentially sensitive behavior, showcasing the spectral peak position as a function of the effective refractive index of the cladding. This trend, as demonstrated later, exhibits excellent agreement with experimental results. Importantly, it indicates that the achievable figure of merit, derived from combining such substantial exponential sensitivity with the intrinsic high‐*Q* factor of the experimental quasi‐BIC, has the potential to significantly improve the limit of detection of the MIP‐BIC sensor, relevant for the clinical scenario mentioned above.

To fully realize the potential of the mechanism, we now explore the detectivity of the MIP‐BIC sensor comparing two different readout strategies, the refractometric spectral shift method with an alternative approach based on the principle of the optical lever.

### 2.2 Evaluation of the Sensing Readout

**Figure 2:**
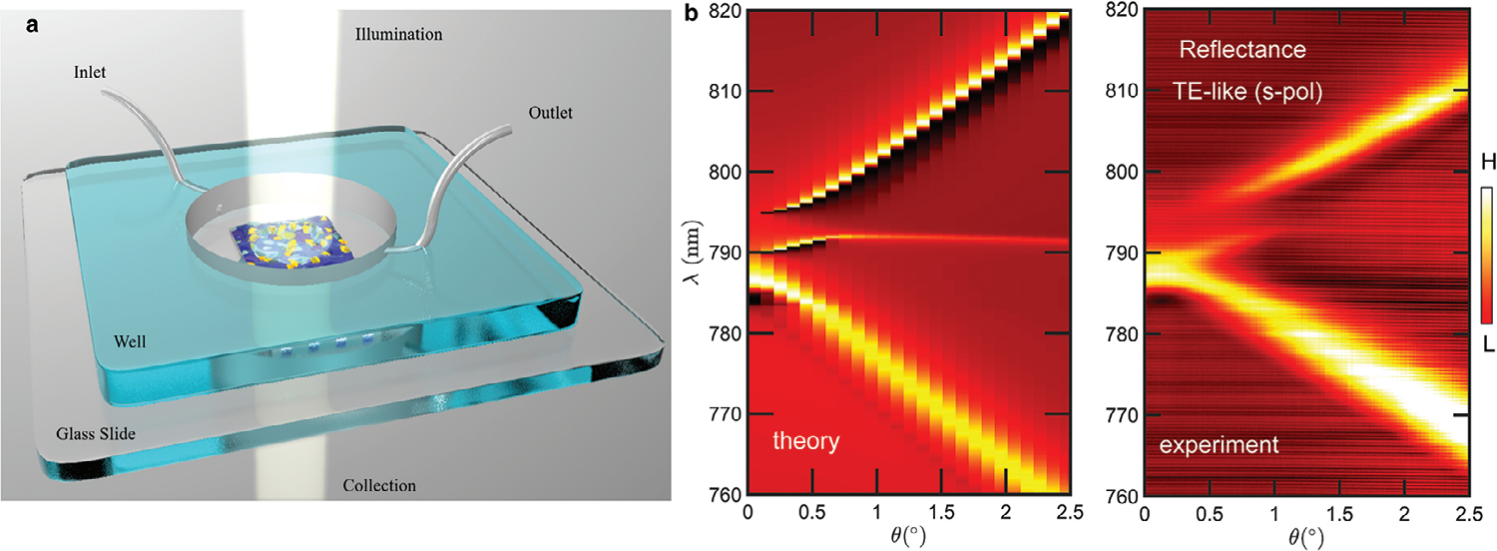
a) Scheme of MIP‐BIC sensor interrogation. b) Simulated reflectance band diagram compared with experimental band diagram of the PhCS for TE‐like modes (s‐polarization) as a function of the incidence angle.

The detectivity of the MIP‐BIC sensor, designed for TGF‐β as outlined previously, was tested through successive incubation of the sensor surface with increasing concentrations of TGF‐β dispersed in a PBS medium. The liquid was injected in the cylindrical sensing region, in volumes of 60 µL for each concentration (**Figure** **2**a). The region hosted the sensitive area of 1 mm^2^ at the bottom. The explored analyte concentration ranged from 0.001 to 10 pM. Figure 2b shows the comparison between calculated and experimental reflectance band diagrams of the nanostructure, revealing an excellent agreement.

Although traceable peak shifts were discerned within 5 min of incubation, the sensogram curve construction involved maintaining the incubation period for typically over 30 min to ensure the saturation of analyte adsorption. After incubation, the MIP‐BIC sensor underwent multiple washes with milli‐Q water to eliminate any excess of non‐specifically adsorbed protein. Subsequently, any residual liquid was expelled to prevent interference in the microfluidic chamber. Full band diagrams were measured at each concentration step to reduce potential systematic errors caused by optical misalignment, vibrations, position shifts, non‐specific protein binding, or liquid interference. This ensures the accuracy of the results. Any detectable shifted peak position was tracked over time for each concentration following rinsing and drying to verify its stability. The stability was tracked by monitoring the statistical fluctuation of the peak intensity and wavelength over time. The measurements were considered stable when the peak fell within one standard deviation of its normal distribution for more than 30 s.

**Figure 3:**
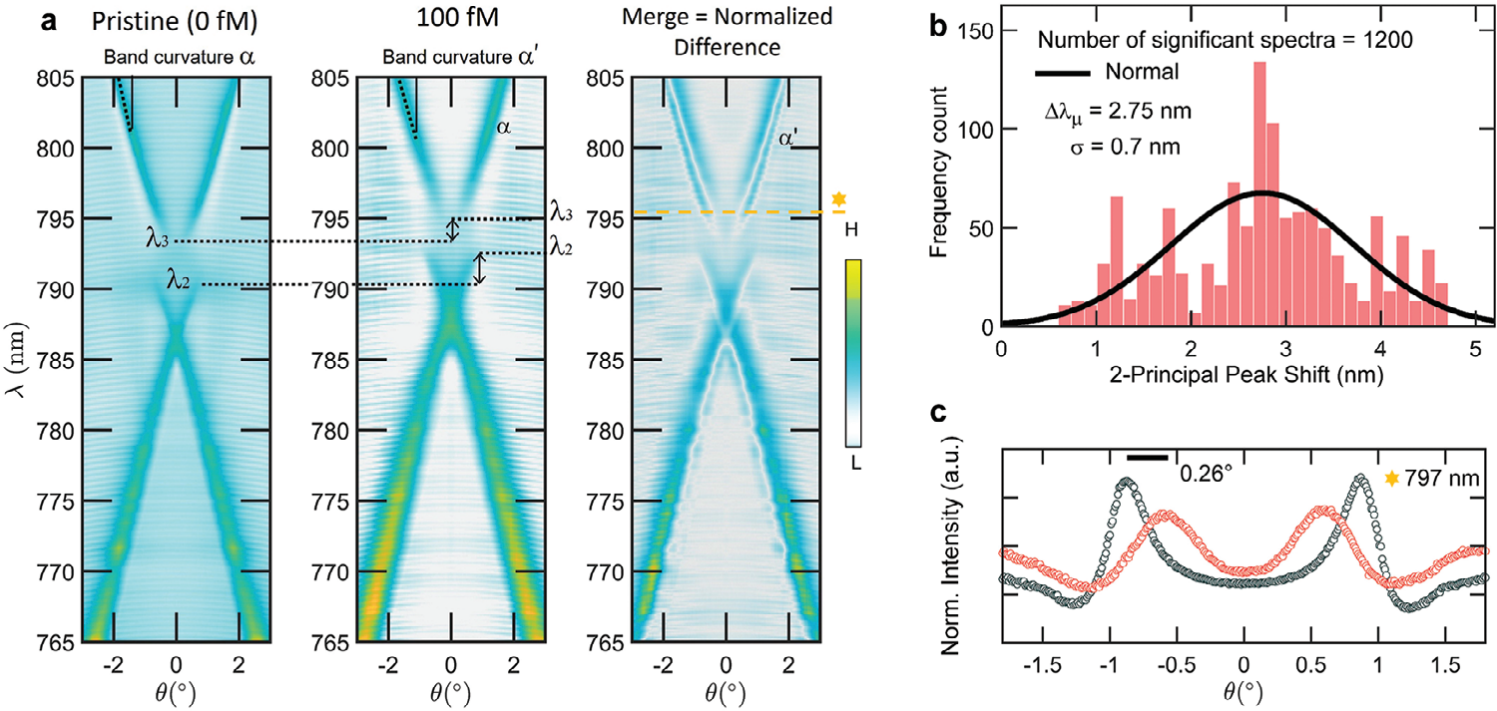
a) Comparison of the reflectance bands before (pristine) and after incubation with TGF‐β concentration *C* = 100 fM, and normalized difference band diagram indicated as merge. b) Representative distribution of the peak shift obtained for *C* = 100 fM for the high‐*Q* factor modes, evaluated for the experimental angles selected as relevant (*N* = 1200 out of 2001 total, range (− 10°, 10°), resolution 0.01°. c) Representative comparison of the angular peak shift before (black dots) and after (red dots) incubation at *C* = 100 fM: the spectral redshift of the modes with the molecular adsorption implies also a shift of the angle at which the spectrum is peaked at a fixed probed wavelength (here 797 nm).

**Figure** **3**a illustrates the system response at *C* = 100 fM and delineates the sensing methodology. The parameter 1 − *T*_*s*_(λ, θ), which closely approximates the reflectance dispersion owing to minimal absorptive losses, is evaluated both before and after incubation. By comparing these measurements, we can discern the variation in the resonance peaks ascribed to the molecular binding, also verifying the stability of the alignment evidenced by the symmetry of the bands around Γ, thus ensuring that any observed shifts are due to the sensing interaction rather than misalignment or other experimental errors. This is important to appreciate tiny perturbations of the peak wavelength associated with ultra‐low concentration of the target molecules. Three following key factors should be considered.

i) The spectral shift between pristine and final peaks at a fixed angle may vary with the monitored incidence angle and depends on the considered dispersion curve (λ_1_, λ_2_, λ_3_). This aspect must be taken into account for analytical quantification. In Figure 3b, a statistical sampling of the spectral shift is presented, considering 1200 angles around **Γ**, resulting in a measurable shift with a mean value of Δλ_µ_ = 2.8 ± 0.7 nm. The standard deviation σ is reduced by a factor of 5, enhancing the accuracy of the response when narrowing the analysis to a region of interest of 1°, sampled with a 0.01° step in the angular scan.
ii) Adsorption of the analyte induces a modification in the band curvature angle α and results in a deterioration of the overall experimental *Q*‐factor of the modes. The concentration‐dependent resonance broadening is more pronounced with higher concentrations. This mechanism holds significant importance; as the analyte concentration decreases to trace perturbations, the *Q*‐factor remains sufficiently large, enabling the detection of minute variations.
iii) The mode redshift induces an additional angular shift when considering the response at a fixed wavelength, stemming from the combined effects of mode energy detuning and band curvature modification. In Figure 3c, the macroscopic angular peak detuning is depicted near the quasi‐BIC wavelength of 797 nm (*C* = 100 fM, detuning of 0.26° = 4.5 mrad). This effect is notable as it can function as an optical lever. The optical lever technique, widely used for measuring the deflection of atomic force microscopy (AFM) cantilevers with high sensitivity,^[^ ^56^ ^]^ employs a probe laser reflection detected by a position‐sensitive photodetector. This approach achieves high sensitivity because even slight angular changes in cantilever deflection (typically below 10 µrad) result in measurable alterations in the laser position on the photodetector. The angular peak detuning of our system can be seen as the laser probe deflection of the optical lever in AFM. The associated deflection is measured in our experiments using a standard goniometer with a resolution of 10^2^µrad.

### 2.3 Evolution of the Sensor Response Around the BICs

**Figure 4:**
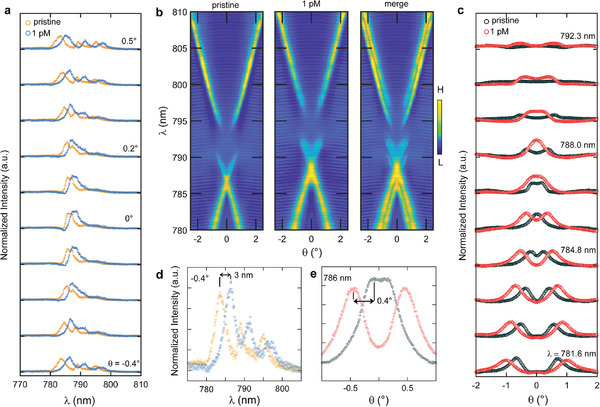
a) Comparison of the evolution of the reflectance spectra (vertical cross‐section of the band diagram) at representative angles near normal incidence at *C* = 1pM. b) Comparison of the full experimental band diagrams highlighting the large variation detected with TGF‐β absorption. c) Horizontal cross‐sections of the band diagram at fixed wavelengths, denoting the angular shift of the peak resonance as a result of the redshift of the resonant modes. d) Zoom of a reflectance spectrum from (a). e) Zoom of a horizontal angular cross‐section from (c).

**Figure** **4**a illustrates the spectral shift following incubation with TGF‐β at *C* = 1 pM, centered around the symmetry point where the quasi‐BICs reside. The complete band diagram is presented in Figure 4b, depicting the angular detuning at representative wavelengths in Figure 4c. Detailed spectral and angular detuning information is extracted from panels Figure 4a,c, respectively, and depicted in Figure 4d,e. The total *Q*‐factor at the quasi‐BIC is 262 when the system is exposed to the target analyte at a concentration *C* = 1 pM.

**Figure 5:**
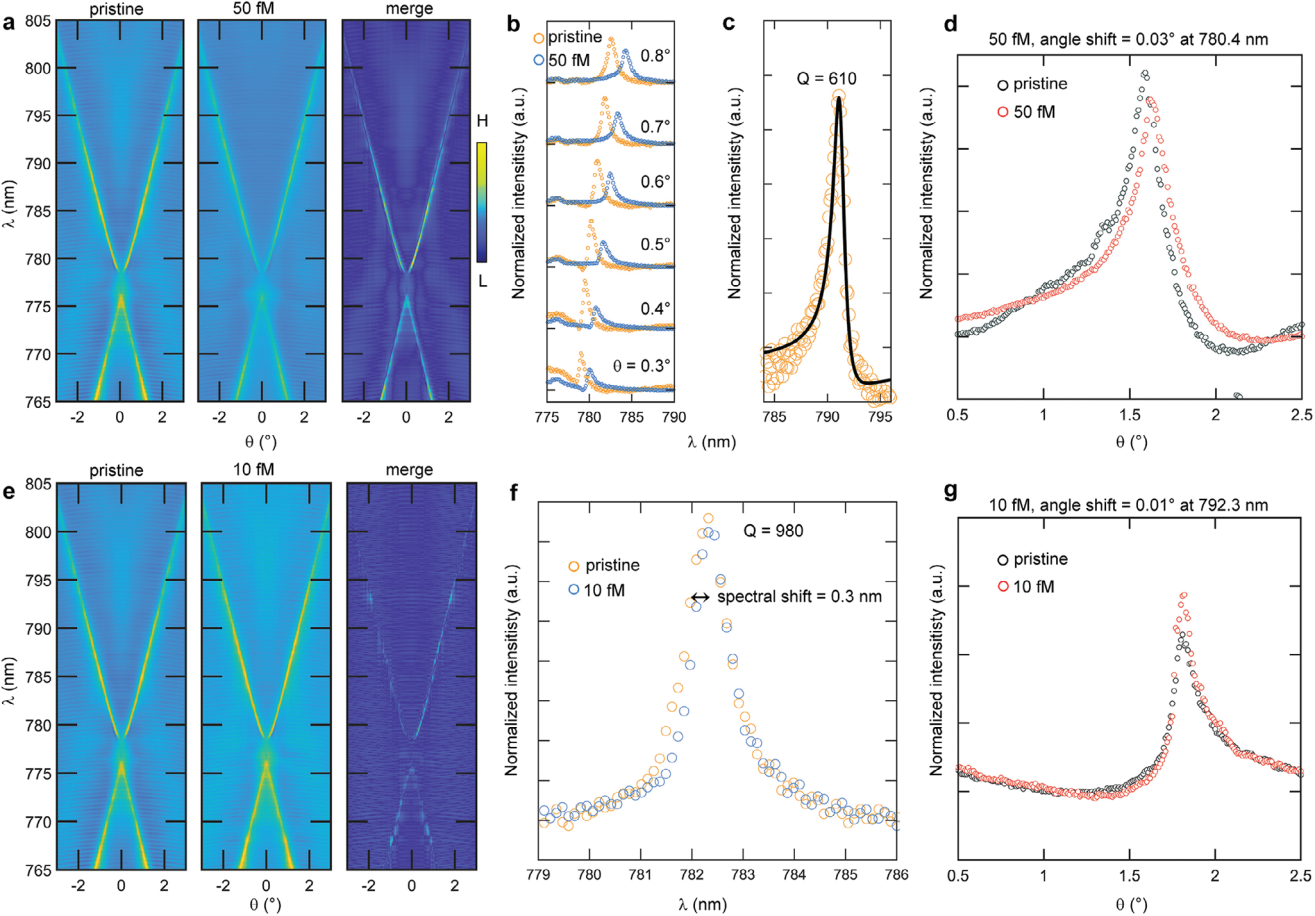
a) Full reflectance TE band diagram comparison at *C* = 50 fM. b) Comparison at representative spectra at fixed angle for *C* = 50 fM, and experimental *Q*‐factor evaluation in (c) fitted as a Fano resonance (black line). d) Representative horizontal angular cross‐section denoting a limited angular peak shift of 0.03°. e) Full reflectance TE band diagram comparison at *C* = 10 fM. f) Comparison of more representative spectra at a fixed angle for *C* = 10 fM, having large experimental *Q*‐factor as less perturbed by the molecular layer, and also pointing out the LOD of the sensor. g) Representative horizontal angular cross‐section denoting the attainment of the LOD also in the angular peak shift, 0.01°.

Two significant measurements are elaborated upon in **Figure** **5**, showcasing the response of the MIP‐BIC sensor to TGF‐β at *C* = 50 fM (Figure 5a–d) and *C* = 10 fM (Figure 5e–g). The combined band diagrams reveal the extent to which it is still possible to discriminate the perturbation‐induced mode detuning in the energy‐momentum space and assess the sensor performance. The LOD is determined by three times the standard deviation of the blank signal. The blank standard deviations were 0.1 nm for spectral readout and 0.01° for angular readout.

Figure 5b highlights how the total experimental *Q* factor near the BIC, within a relatively large range of angles around the high‐symmetry point, provides valuable spectral information to quantify the minute perturbation induced at 50 fM precisely. The results of the Fano resonance fit, yielding *Q* = 610, are reported in Figure 5c. On the other hand, Figure 5d emphasizes that at this concentration, the LOD based solely on angular detuning scan is approaching, with a shift of 0.03°. In contrast, the LOD measured by considering spectral detuning at a fixed angle is achieved only at the much lower *C* = 10 fM, as depicted in Figure 5e–f. This superior detectivity is ensured by the large *Q* = 980.

While relying solely on angular detuning may not yield accurate detection at 10 fM using the angular peak position obtained through the goniometer scan (Figure 5g), it is noteworthy that enhancing detectivity could be achieved through the exploration of alternative strategies for peak position tracking. These strategies could potentially overcome limitations posed by the goniometer resolution and our basic measurements, thereby improving overall performance, for instance using a position‐sensitive photodetector.

In this work, however, our primary focus remains on applying the MIP‐BIC for detecting TGF‐β at physiological levels in potential patients. The emphasis lies in achieving high‐precision discrimination at concentrations higher by several orders of magnitude, specifically in the 100‐pM range. Notably, in saliva, the detected levels of TGF‐β in patients average at 24.1 (7–87.9) ngmL^−1^, compared to control levels of 14.8 (5.4–30.1) ngmL^−1^.^[^ ^5^ ^]^ These values are relatively close to each other, highlighting the critical importance of accurate detection to validate any sensing strategy. In this scenario, the substantial potential of the angular peak readout will become evident. The subsequent section explores this in‐depth analysis, elucidating the effectiveness of this method in providing accurate and reliable results for detecting TGF‐β at concentrations relevant to potential patient scenarios.

### 2.4 Determination of TGF‐β in Saliva Samples and Discussion

**Figure 6:**
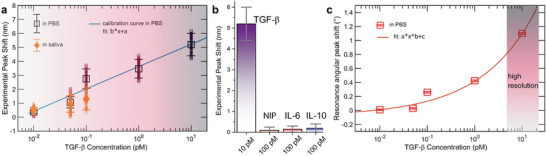
a) Calibration of the spectral peak response as a function of the analyte concentration. Square points are experimental points of TGF‐β in PBS medium. Diamond points are measurements carried out in spiked saliva and projected on the calibration curve. For each concentration, the whole pool of spectral data, over the selected 1200 angles of sampling, is reported together with the associated mean value and standard deviation. The error bars represent the standard deviation of the distributions of peak shifts, shown in detail in Figure S4 (Supporting Information). The solid line is the linear fit of the calibration. b) Specificity of the MIP‐BIC response: a positive variation over the LOD threshold is present at the physiological level of 10 pM of TGF‐β in saliva, while for 100 pM of competitive molecules (IL‐6 and IL‐10) no signal is detected. The control NIP‐BIC platform, insensitive to TGF‐*beta*, is also exposed to 100 pM of TGF‐β with no response. The error bars represent the standard deviation of the peak shift distribution for MIP measurements of TGF‐β, whereas they represent the maximum instrumental error for the control measurements on NIP, IL‐6 and IL‐10, assumed as the spectrometer resolution of 0.25 nm. c) Experimental angular peak calibration with analyte concentration in PBS medium: the experimental angular peak shift provides an exponentially sensitive readout with high‐resolution capability in the most relevant range of physiological levels, where resolving with high precision TGF‐β concentration is crucial to discriminate between normal and abnormal levels. The error bars of ±0.01° correspond ±2 × α, where α is the accuracy of goniometer positioning system (α = ±0.005°) provided by the manufacturer.

The calibration curve established using measurements conducted in a PBS medium is shown in **Figure** **6**a. As reported in a previous study,^[^ ^29^ ^]^ the BIC bulk sensitivity varies exponentially with the cladding refractive index. This behavior was confirmed in the simulations included in Figure 1c,e for sufficiently large cladding thicknesses. However, for a top nanoscale thickness cladding, the spectral shift increase could be approximately considered linear. A linear regression is indeed used in Figure 6a. Moreover, Figure S3a (Supporting Information) reports the *Q* as a function of the TGF‐β concentration.

The experimental *Q*‐factor consistently falls below the calculated value based on an ideal infinite geometry, owing to finite sample leakage, scattering losses due to local geometric imperfections, dielectric fluctuations, and losses induced by the inhomogeneities of the MIP film, as well as scattering from molecular adsorbates. The increase in the analyte concentration perturbs the system thus deteriorating the *Q* value. This conclusion arises from the observed damping of the *Q*‐factor as molecular adsorption in the MIP increases (Figure S3a, Supporting Information). However, the larger energy detuning of the mode compensates for this, ensuring that the peaks remain distinguishable. In Figure S3b (Supporting Information), the experimental figure of merit (FOM) as a function of the TGF‐β concentration is also reported. FOM, here denoted as Δλ_µ_ *Q* represents a comprehensive metric of the performance of our device and exhibits a maximum value at around 100 fM, where spectral shift and *Q* are large enough to guarantee the high accuracy of the measurements. Error bars represent the standard deviations obtained by considering the full scanned range of angles. Peak analyses are shown in Figure S4 (Supporting Information). For evaluating the response of the sensor, we estimated the local sensitivity of the nonlinear sensogram curve reported in Figure 6a, estimated according to ref. [29] and defined as $s=\frac{\partial \;\Delta \lambda_{\mu }}{\partial \;C\;}$, in particular at the maximum FOM concentration *C* = 100 fM. The estimation gave *s* = 1.4 × 10^−2^ nmfM^−1^.

To showcase the practical utility of the developed MIP‐BIC‐based sensor and assess its performance in a complex matrix simulating a real diagnostic setting, we employed the sensor for detecting target molecules in spiked saliva samples. Rebinding incubation was conducted with varying concentrations in artificial saliva, spanning from 10 to 100 fM. The outcomes are depicted in Figure 6a and correlated with the calibration curve. To evaluate the affinity and selectivity of the synthetic receptor for the template molecule, we designed two parallel PhCS setups. The negative control group was exposed to two different cytokines, specifically interleukin‐6 (IL‐6) and interleukin‐10 (IL‐10), separately (Figure 6b). The MIP with TGF‐β underwent rinsing using the previously established protocol to eliminate the template molecule. Subsequently, it was incubated with a buffer solution containing interfering molecules at a concentration of 100 pM. The spectra obtained after incubation with IL‐6 and IL‐10 showed no significant differences compared to the bare PhCS, indicating the absence of specific interactions between IL‐6 and IL‐10 and the binding cavities in the polymer. The cross‐reactivity values, calculated as the ratio between the non‐specific interleukins and MIP peak shift responses, showed a value of 5% for both IL‐6 and IL‐10, thus suggesting really poor interactions with the MIP film of the non‐specific molecules.

A supplementary negative control involved the assessment of a non‐imprinted polymer (NIP) deposited using the same protocol. After washing with an EtOH/H_2_O and NaOH solution, a concentration of 100 pM of TGF‐β was incubated on the NIP. No specific binding between the protein and NIP was detected, consistent with the absence of available binding sites in the NIP (Figure 6b). Also, 10 pM of TGF‐β was incubated on the NIP to evaluate the imprinting factor. The ratio of experimental peak shifts of TGF‐β on MIP and NIP, respectively, was evaluated resulting in an imprinting factor of 52. The dissociation constant *K*_*D*_ estimated in Supporting Information reveals a remarkable value of 125 ± 45 fM for the MIP‐BIC sensor, which indicates an excellent affinity of the imprinting sites for TGF‐β.^[^ ^57^ ^]^ Details of the calculation are reported in Figure S9 (Supporting Information), considering the average values from five samples per concentration.

For context, it is helpful to consider the levels of TGF‐β typically found in saliva. Research indicates that TGF‐β concentrations range from 7 to 87.9 ngmL^−1^ in patients, with an average of 24.1 ngmL^−1^, compared to 5.4–30.1 ngmL^−1^ in controls, with an average of 14.8 ngmL^−1^.^[^ ^5^ ^]^ Thus the minimum of the range of interest can be considered above 100 pM, or 5 ngmL^−1^. Given these overlapping concentration ranges between normal and patient levels, accurate differentiation is crucial for clinical diagnosis and treatment monitoring. The sensor must not only be highly sensitive but also capable of resolving small differences in concentration with high precision.

Let us consider this in detail, comparing the two sensing readouts. In Figure 6a, the peak shift readout shows the sensor achieving maximum performance at an extremely low concentration of 0.1 pM (0.004 ngmL^−1^), which is three orders of magnitude lower than the 5 ng/mL threshold considered clinically relevant.^[^ ^5^ ^]^ This high sensitivity allows the sensor to effectively screen for several tens of biomarkers, using very small volumes from the same patient's sample, 100‐fold reduced instance, thereby increasing the statistical accuracy of screening a full range of biomarkers. This capability is particularly advantageous in clinical settings where precise and early detection of biomarkers at very low concentrations can lead to better diagnosis and monitoring of diseases. The ability to operate efficiently with minimal sample volumes also reduces the biological sample requirements, making the process less invasive.

Figure 6c depicts the angular peak shift detected across the same range of analyte concentrations. Upon closer examination of its behavior, it becomes evident that the exponential sensitivity of the evanescent field MIP‐BIC sensor is more pronounced in this case. Notably, there is a remarkable angular shift of over 1° at the level of 10 pM (0.4 ngmL^−1^), highlighting the sensor capability for precise quantification of TGF‐β in this range. In particular, we can estimate a conservative angular resolution of 0.05° (i.e., tenfold the angular positioning accuracy) for the angular shift, which translates into a conservative estimation of the concentration resolution of 0.5 pM (0.02 ngmL^−1^) in the range of maximum slope in Figure 6c. In this case, the sensor can be exposed to a patient's sample diluted by a factor of 10, still demonstrating a resolution two orders of magnitude greater than what is needed to distinguish between normal and abnormal TGF‐β levels. This not only enables accurate and specific quantification but allows also this angular readout to test simultaneously the same sample against tens of other biomarkers, which increases the capability of correctly scoring the case under study.

Therefore, comparing the two sensing readouts, we can conclude that the higher‐sensitive spectral shift is more valuable in trace‐detection schemes where ultra‐low concentration must be estimated, whereas the angular readout can be used to devise a sensor for routine applications, using the large resolution to more precisely estimate the concentration level.

To contextualize our findings, **Table** **1** presents a comparison of the MIP‐BIC sensing platform in the broader landscape of TGF‐β detection technologies. This table collates key performance metrics such as the limit of detection and detection range, offering a direct comparison that highlights the advantages and potential limitations of each sensor technology. Other primary sensing techniques are based on enzyme‐linked immunosorbent assay (ELISA), surface plasmon resonance (SPR), and electrochemical biosensors.

**Table 1:** Comparison between MIP‐BIC‐based device and other biosensors for the detection of TGF‐β.

Reference	LOD	Detection range	Detection methods	Biorecognition element	Sample
[53]	0.95 pgmL^−1^	2.5‐1000 pgmL^−1^	Amperometry	Antibodies	Saliva
[58]	211 pM	0‐300 pM	Fluorescence	Antibodies	Culture medium
[59]	0.570 ngmL^−1^	1–1000 ngmL^−1^	Impedance	Antibodies	Clinical serum
[60]	0.09 ngmL^−1^	0.5‐20 ngmL^−1^	Voltammetry	MIP	Saliva
[61]	10 pgmL^−1^	15‐3000 pgmL^−1^	Amperometry	Antibodies	Urine
[62]	0.2 nM	1‐50 nM	Electrochemical	Cas 12a cr‐RNA	Protein sample
[63]	1.3 pM	0.1‐10000 ngmL^−1^	Optical	Gold Nanoprobe	Culture medium
[64]	–	0‐20 ngmL^−1^	Optical	PDMS sheet	Breast cancer cell
This work*	10 fM (0.48 pgmL^−1^)*	0.001‐10 pM	Optical	MIP	Saliva

The advantages of BIC‐enhanced MIP include high specificity due to tailor‐made binding sites, robustness and stability in various environmental conditions, and the potential for miniaturization and integration into portable devices. Although it may require extensive optimization to achieve high sensitivity, our BIC‐enhanced sensing provides sensitivity beyond the current state‐of‐the‐art.

ELISA is based on antibody‐antigen interactions, where TGF‐beta is captured by specific antibodies and detected using an enzyme‐linked secondary antibody. Thus, ELISA requires multiple steps, is time‐consuming and relatively expensive due to the cost of antibodies and reagents. It requires large sample volumes, and lacks multiplexing capabilities, hindering its utility in rapid diagnostics. The signal is a fluorescence intensity not unequivocally proportional to the target concentration, which limits its quantification accuracy. Our approach based on a label‐free refractometric shift overcomes these limitations.

Similar to our BIC‐enhanced MIP, SPR it is a real‐time and label‐free detection method, providing high sensitivity and the ability to study binding kinetics. However, SPR surface regeneration can be challenging, affecting reproducibility, while our dielectric material approach does not suffer from this limitation.

Finally, electrochemical biosensors utilize electrodes modified with biorecognition elements (such as antibodies, aptamers, or peptides) that interact with TGF‐beta, leading to measurable electrochemical signals. These sensors offer high sensitivity and rapid response times, cost‐effective, and simple instrumentation, but time‐consuming, complex, and costly material preparation. Our approach is based on a more simple architecture, still proving competitive sensitivity, as evidenced in Table 1.

## 3 Conclusion

In conclusion, our study introduces a novel approach that combines a large‐area photonic crystal slab capable of sustaining bound states in the continuum with a molecularly imprinted polymer tailored for specific recognition of TGF‐β. BICs emerging by breaking Dirac cone dispersion provide the platform for enhancing light‐matter interactions at imprinted sites of MIP. The breakthrough resides in the synergistic interplay of field overlap specificity and selectivity guided by the MIP. In essence, our work introduces an architecture that streamlines the detection process, eliminating the need for complex sandwich immunoassays. We explore the application of this hybrid system for oral cancer biomarker sensing in artificial saliva, leveraging its non‐invasive collection method and accessibility. Our extensive analysis demonstrates the large sensitivity and accuracy of the MIP‐BIC sensor in quantifying TGF‐β. Notably, the sensor exhibits exponential sensitivity also in the angular shift readout, of great potential even at low physiological concentrations for high‐precision quantification. We demonstrate a limit of detection of 10 fM with high resolution in spiked saliva, surpassing existing state‐of‐the‐art methods. The successful integration of the highly sensitive BIC sensor and the MIP specificity positions makes our MIP‐BIC sensor a scalable sensing technology for ultrasensitive biosensing in point‐of‐care diagnostics and various other applications. The limit of detection is linked to the quality factor of the mode. When the patterned area of the photonic crystal slab is decreased aiming at miniaturizing the sensing area, the *Q*‐factor tends to diminish,^[^ ^26^ ^]^ thereby imposing a limitation on the LOD. This reduction in the *Q*‐factor can be attributed to larger leakage of light from the sample. Selecting materials with higher refractive indices, such as silicon, it should also become feasible to mitigate the effects of reduced patterned areas.

## 4 Experimental Section

### Numerical Simulations and Fabrication

Numerical simulations of the transmittance spectra of the PhCSs were conducted using a comprehensive 3D rigorous coupled‐wave approach (RCWA) based on a Fourier modal expansion. Ansys Lumerical 2023 was employed for these simulations. Additional finite element method‐based simulations were performed using Comsol Multiphysics 6.0. Bloch periodic boundary conditions were applied along the *x*‐ and *y*‐directions, while perfectly matched‐layer boundary conditions were enforced on the top and bottom surfaces normal to the *z*‐direction. Mesh refinement along the *z*‐axis was implemented with a step size of 1 nm within the smallest PhCS regions, gradually increasing to 20 nm away from the structure. The PhCS sensor was constructed on a square lattice of cylindrical holes in silicon nitride (Si_3_N_4_) deposited on a SiO_2_ substrate through plasma‐enhanced chemical vapor deposition (PECVD). The design was patterned using standard high‐voltage electron beam lithography.^[^ ^39^ ^]^ Geometric parameters of the square lattice were optimized to excite the BIC resonances in the 800 nm range, specifically with a lattice constant *a* = 540 nm, hole radius *r* = 144 nm, experimental Si_3_N_4_ slab thickness *t* = 90 nm (variable in simulation), and conformal MIP imprinting thickness δ*t* = 25 nm (variable in simulation). The silicon nitride dispersion utilized in simulations was experimentally measured through variable angle ellipsometry (Figure S5, Supporting Information).

### TGF‐β‐Binding MIP: Synthesis and Deposition

Following a previously reported experimental procedure,^[^ ^65^ ^]^ polydopamine films were self‐polymerized on the PhCS by immersing it in a solution of 0.1 mgmL^−1^ dopamine hydrochloride in Tris‐HCl overnight. Before polymerization, TGF‐β was introduced into the dopamine solution as a template molecule at a concentration of 1 µgmL^−1^. After polymerization, the modified BIC sensor underwent washing with a solution of EtOH/H_2_O and NaOH 0.25 M (2:1) to elute the template. A schematic diagram illustrating the MIP sensor fabrication is provided in Figure S6 (Supporting Information). In mildly basic solutions Dopamine (DA) could undergo oxidation generating chemical species that emulate the composition of adhesive proteins in mussels, serving as a building block for the spontaneous deposition of thin polymer films onto a diverse range of materials, including metals, oxides, polymers, semiconductors, and ceramics.^[^ ^47^ ^]^ To exert precise control over all stages of MIP production (synthesis, template removal, and analyte rebinding), the same protocol was executed on a platinum (Pt) electrode, and the entire process was electrochemically characterized (see Supporting Information). As detailed in ref. ^[^ ^47^ ^]^, dopamine could indeed form thin films on both platinum and silicon nitride with similar characteristics in terms of composition and homogeneity.

To construct the detector calibration curve, TGF‐β at various concentrations was dispersed in a PBS medium. Subsequently, artificial saliva (NeutraSal) was prepared by dissolving a foil sachet in 30 mL of Milli‐Q water and utilized as a buffer for TGF‐β.

### MIP‐BIC Sensor Characterization

All the MIP‐BIC sensors were morphologically and optically characterized before and after MIP deposition. An AFM was used to investigate the surface topography of the polymeric layers, revealing a PDA layer in the range of 10–25 nm (Figure S7, Supporting Information). A PDMS (polydimethylsiloxane) chamber was integrated into the device in order to infiltrate the analyte. A sketch of the final device was shown in Figure S8 (Supporting Information).

The spectral position and mode quality factors were determined through angle‐resolved transmittance measurements using a light source from NKT Photonics (SuperK EXTREME) with an emission range of 400–2400 nm, featuring single‐mode output and a maximum power of 4 W. This methodology aligns with established procedures.^[^ ^38^ ^]^ Reciprocal space dispersion was measured by rotating the sample with an angular step of 0.01° using an externally driven system. A high‐resolution spectrometer (Ocean Optics HR4000) was employed for precise spectral detection. The entire process was automated through a customized MathWorks MATLAB routine for data acquisition and band reconstruction. This routine includes a filtering step through a fast Fourier transform to eliminate background noise and real‐time tracking of the main peak positions throughout the entire angular range of inspection.

### Additional Comments on Dirac Cone Breaking

In a 3D PhC slab structure, each mode could be distinguished by its predominant TE‐like or TM‐like character. The spatial configurations assumed by the optical field decrease with the thickness of the slab *t*, which favors their coalescence. In Section 'Evolution of the dispersion bands with the PhCS thickness' in the Supporting Information, and Figures S10–S12 (Supporting Information), more physical insight was provided into the mechanism of band splitting and BIC formation.

## Conflict of Interest

The authors declare no conflict of interest.

## Supporting information

Supporting Information

## Data Availability

The data that support the findings of this study are available from the corresponding author upon reasonable request.
