# Supplementary material for: Molecularly Imprinted Polymer Sensor Empowered by Bound States in the Continuum for Selective Trace‐Detection of TGF‐beta
Source: Adv Sci (Weinh). 2024 Sep 5;11(41):2401843. doi: 10.1002/advs.202401843 (PMC11538715; doi:10.1002/advs.202401843)
Supplement: Supplementary file 1 — Supporting Information [file ADVS-11-2401843-s001.pdf]

ADVANCED SCIENCE
Open Access

Supporting Information

for *Adv. Sci.*, DOI 10.1002/advs.202401843

Molecularly Imprinted Polymer Sensor Empowered by Bound States in the Continuum for Selective Trace-Detection of TGF-beta

Gianluigi Zito, Giulia Siciliano, Aida Seifalinezhad, Bruno Miranda, Vittorino Lanzio, Adam Schwartzberg, Giuseppe Gigli, Antonio Turco, Ivo Rendina, Vito Mocella, Elisabetta Primiceri and Silvia Romano**

Supporting Information

Molecularly Imprinted Polymer Sensor Empowered by Bound States in the Continuum for Selective Trace-Detection of TGF-beta

Gianluigi Zito† *Giulia Siciliano*† *Aida Seifalinezhad* *Bruno Miranda* *Vittorino Lanzio* *Adam Schwartzberg* *Giuseppe Gigli* *Antonio Turco* *Ivo Rendina* *Vito Mocella* *Elisabetta Primiceri** *Silvia Romano**

G. Zito, A.Seifalinezhad, B. Miranda, I. Rendina, V. Mocella, S. Romano
Institute of Applied Sciences and Intelligent Systems, National Research Council
Via Pietro Castellino 111, Napoli, 80131, Italy
G. Siciliano, A. Turco, G. Gigli, E. Primiceri
Institute of Nanotechnology, National Research Council
c/o Campus Ecotekne, Via Monteroni, Lecce, 73100, Italy
V. Lanzio, A. Schwartzberg
Molecular Foundry, Lawrence Berkeley National Laboratory
1 Cyclotron Rd, Berkeley, 94720, California
elisabetta.primiceri@cnr.it silvia.romano@cnr.it
† The authors equally contributed to this paper.

Keywords: *bound states in the continuum, molecularly imprinted polymer, biosensing, cytokine*

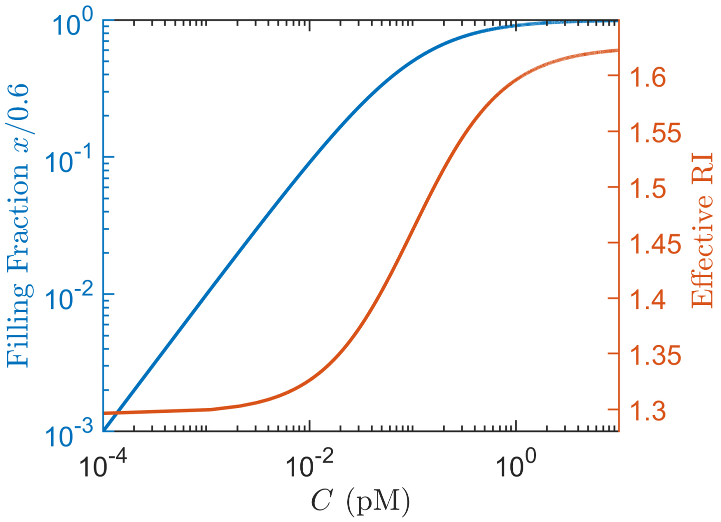

Figure S1: Langmuir isotherm (blue solid line) and retrieved behavior of the effective refractive index (RI) as function of analyte's concentration.

Effective refractive index model for TGF-β adsorption. The investigation into the dependence of the BIC sensor on the refractive index of the Molecularly Imprinted Polymer (MIP) layer was conducted as follows. The refractive index of the MIP under conditions of high density and uniformity is denoted as $n_{\mathrm{MIP}} = 1.74$. A Langmuir isotherm, representing the surface binding process with a maximal filling fraction of $0.6x$ (equivalent to a 60% filling fraction in random close packing), is considered. This filling fraction is proportional to the molecule concentration C in the solvent and is expressed as:

$$x = \frac{kC}{(1 + kC)}, \quad (1)$$

where k is the dissociation constant. To model the binding of analyte molecules, an effective refractive index $n_e(C)$ is introduced to account for the conformal MIP layer. A MIP film with voids, created by template molecules, is envisaged. These voids are expected to be filled with TGF-β upon subsequent resorption.

The asymptotic adsorption level is described by the MIP being fully filled with molecules, forming an approximate monolayer. An upper limit for the TGF-β-MIP filling fraction of 60% is anticipated, as mentioned earlier. Consequently, the resorption of TGF-β into MIP is simulated by varying the effective refractive index of the cladding, denoted as n_e, in the range from 1.3 (corresponding to a MIP matrix with 60% voids) to 1.6. This upper limit corresponds to a MIP matrix with 60% filled with TGF-β of refractive index $n_{\mathrm{tgf}} = 1.5$. The relationship is given by:

$$n_e = (1 - 0.6)n_{\mathrm{MIP}} + 0.6\left[n_{\mathrm{tgf}}x + (1 - x)\right], \quad (2)$$

which can be further expressed, using Eq. (1), as a function of the concentration C and parameterized based on the actual value of k. **Figure S1** illustrates the evolution of $n_e(C)$ with TGF-β concentration.

The progression of the maximum achievable shift resulting from complete adsorption of TGF-β in the MIP film was assessed in relation to its thickness δt and is depicted in **Figure S2**. Notably, at a thickness of 25 nm, the anticipated shift is approximately 6.5 nm. This aligns remarkably well with the calibration curve experimentally measured and presented in Figure 6a of the main text.

Modification of the quasi-BIC quality factor vs concentration. The ability to discern minute variations in the spectral peak position of a quasi-BIC resonance is directly linked to its high Q-factor. This high Q-factor enables the identification of distinct spectral profiles of Fano type, even when partially overlapped, with translated maxima and broadened full width half maximum due to the monitored

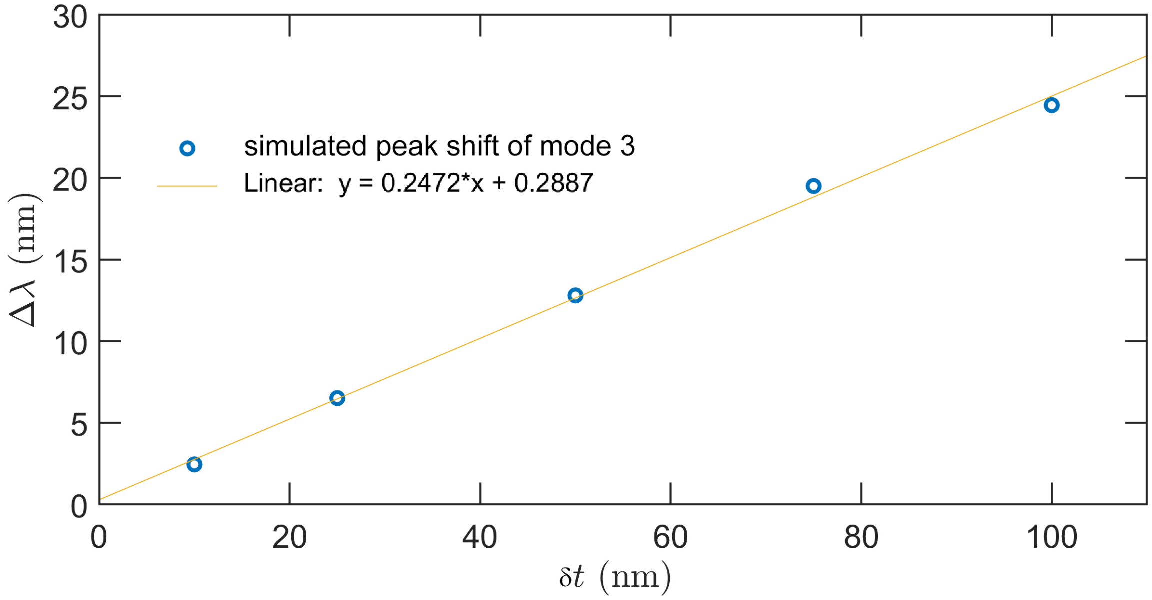

Figure S2: The spectral peak shift $\Delta\lambda$ of mode λ_3 is examined as a function of δt for $n_e(C) = 1.6$, considering a PhCS with parameters $a = 540$ nm, $t = 93$ nm, and $r = 144$ nm.

perturbation of the system. The MIP-BIC sensor leverages a quasi-BIC resonance with an exceptionally large Q-factor, particularly in proximity to the symmetry point $\mathbf{\Gamma}$. Consequently, as the perturbation is progressively removed with decreasing analyte concentration, the Q-factor increases towards its original unperturbed value. With the increasing analyte concentration perturbing the system, the Q-factor deteriorates. However, the larger energy detuning of the mode compensates for this, ensuring that the peaks remain distinguishable. The experimental evolution of the Q-factor as a function of analyte concentration is depicted in **Figure S3a**. In **Figure S3b**, we present the figure of merit denoted as $\Delta\lambda_\mu Q$. This metric serves as a comprehensive measure, reflecting the system's performance by amalgamating both substantial peak shift and a high-quality factor.

Peak shift analysis. In **Figure S4**, the statistical distribution of the quasi-BIC peak shift is depicted. The spectral shift is measured as a function of concentration C across a range of selected spectra, denoted as N. The number of spectra varies from 25 to 1200, depending on the spectral information derived from the full band diagrams acquired after exposing the MIP-BIC sensor to different concentrations C of the analyte.

Simulations. In **Figure S5**, the refractive index dispersion of the silicon nitride film employed in the production of photonic crystal slabs is reported, showcasing both the real part n_1 and the imaginary part n_2. This dispersion is crucial for the optical response calculations in Lumerical RCWA. Notably, the behavior of the imaginary part indicates the absence of detectable absorption beyond $n_2 = 10^{-6}$ in the infrared frequency range.

The fitted dispersion used in simulations converges to $n_1 = 2.06178$ at 800 nm, with a tolerance of 0.001 and a maximum of 6 coefficients. Simultaneously, the imaginary part n_2 stabilizes at 2×10^{-4} with an imaginary weight of 100 with fitting.

MIP deposition and characterization. The Pt electrode surface underwent polishing with 0.3 and 0.05 μm wet alumina slurry, followed by cleaning with Milli-Q water. Subsequently, the electrode underwent cyclic potential sweeps between -0.2 and 1.0 V vs Ag/AgCl in 0.5 M H_2SO_4 until a stable cyclic voltammogram was achieved. Electrochemical measurements employed an Autolab potentiostat (PGSTAT 204, Metrohm), using a standard three-electrode configuration with a platinum disk and wire as working and counter electrodes, respectively, and an Ag/AgCl (3M KCl) electrode as a reference. To optimize

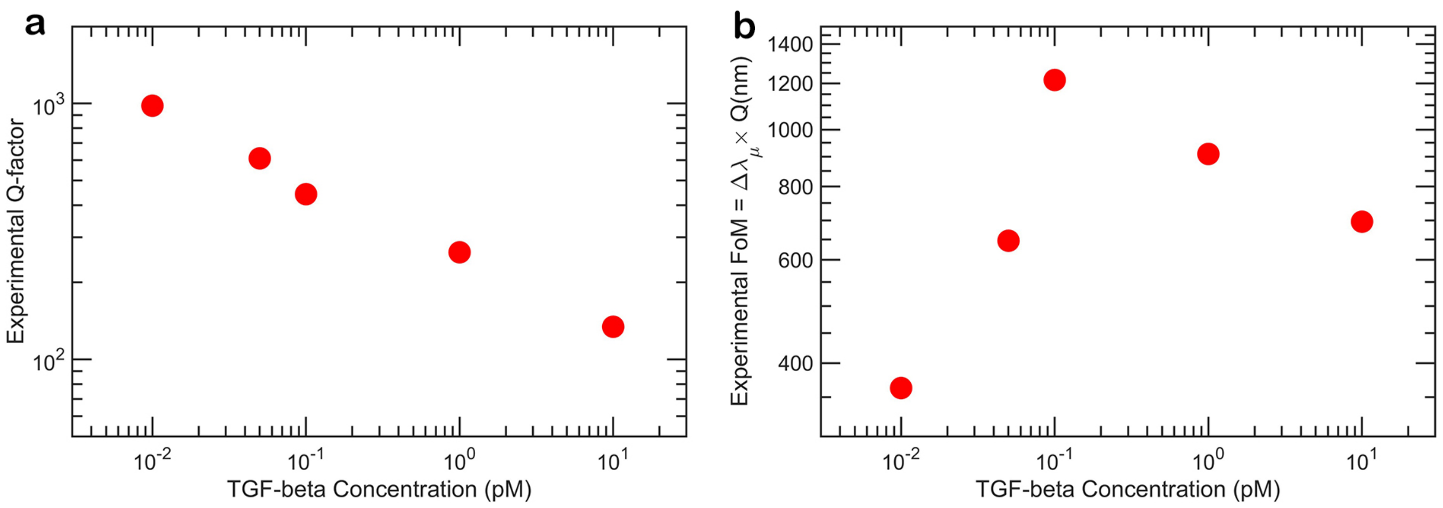

Figure S3: (a) Experimental Q-factor from $\Delta\lambda_\mu(C)$ vs TGF-β concentration. (b) Experimental figure of merit (FoM) defined as $\Delta\lambda_\mu Q$, indicating the maximum accuracy of the system in the range 100 fM.

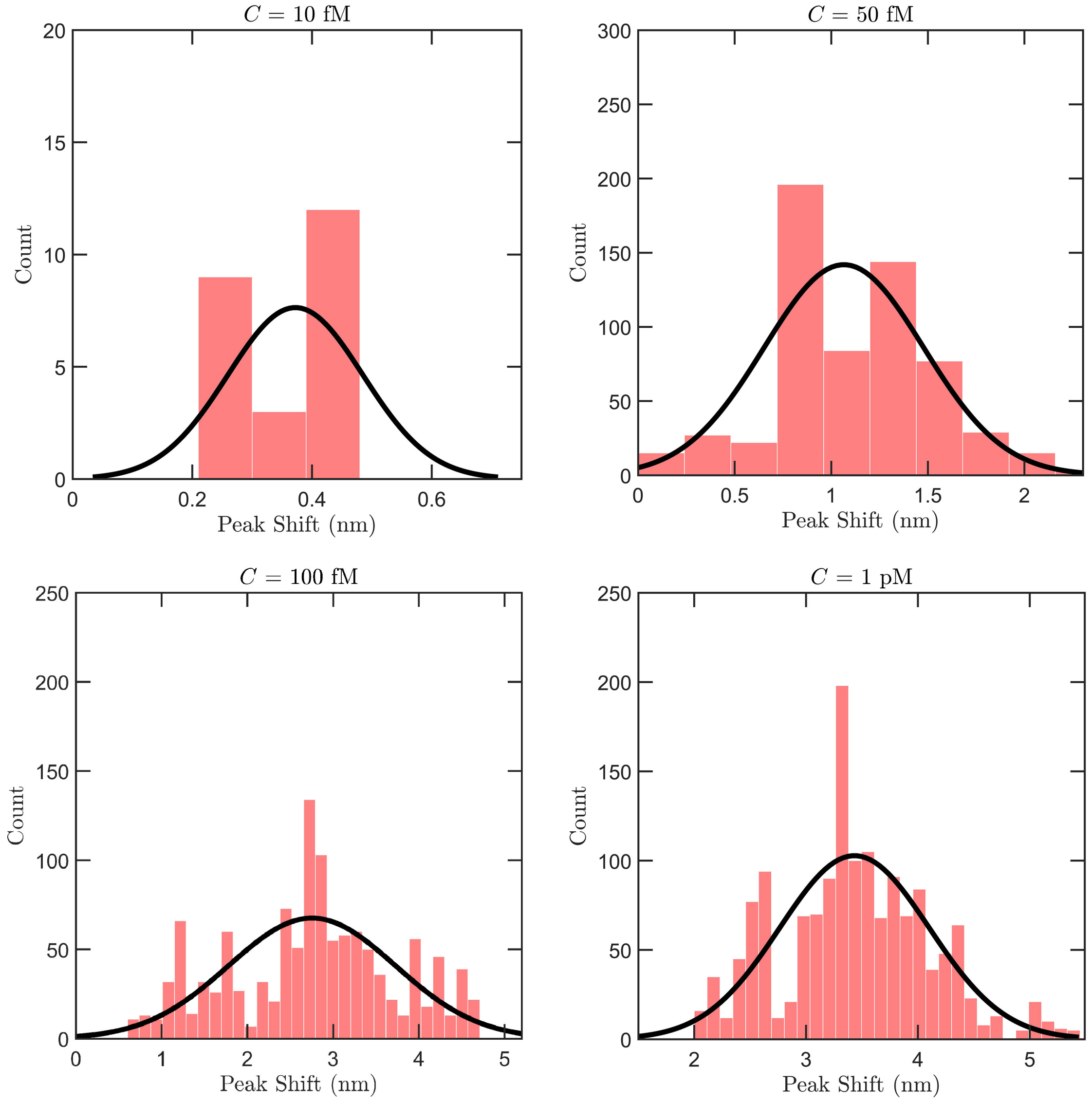

Figure S4: Statistical distribution of the PhCS peak shift measured for the quasi-BIC modes, detected as function of concentration C.

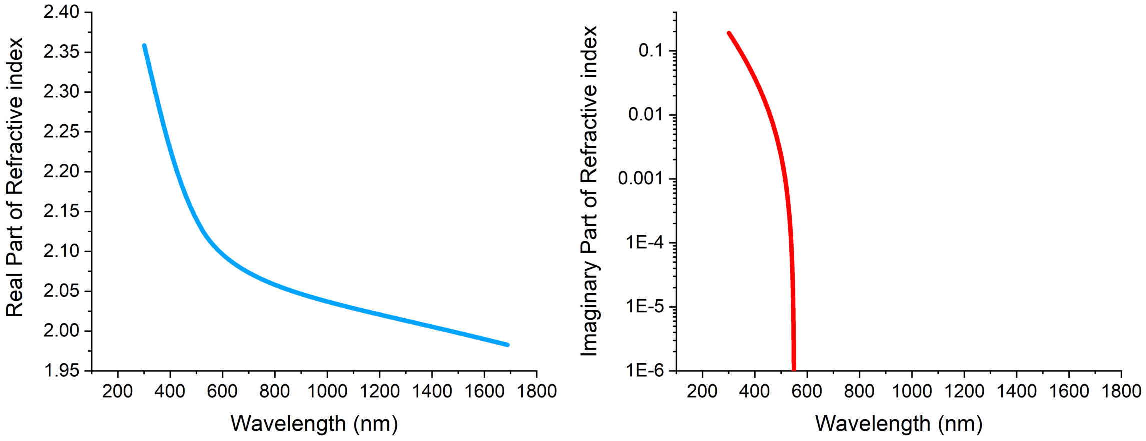

Figure S5: Refractive index dispersion of the fabricated unpatterned silicon nitride film measured by variable angle ellipsometric spectroscopy (UVISEL Plus, Horiba Jobin Yvon, Spectroscopic Ellipsometer), used for numerical simulations (Lumerical 2023 R1, RCWA module).

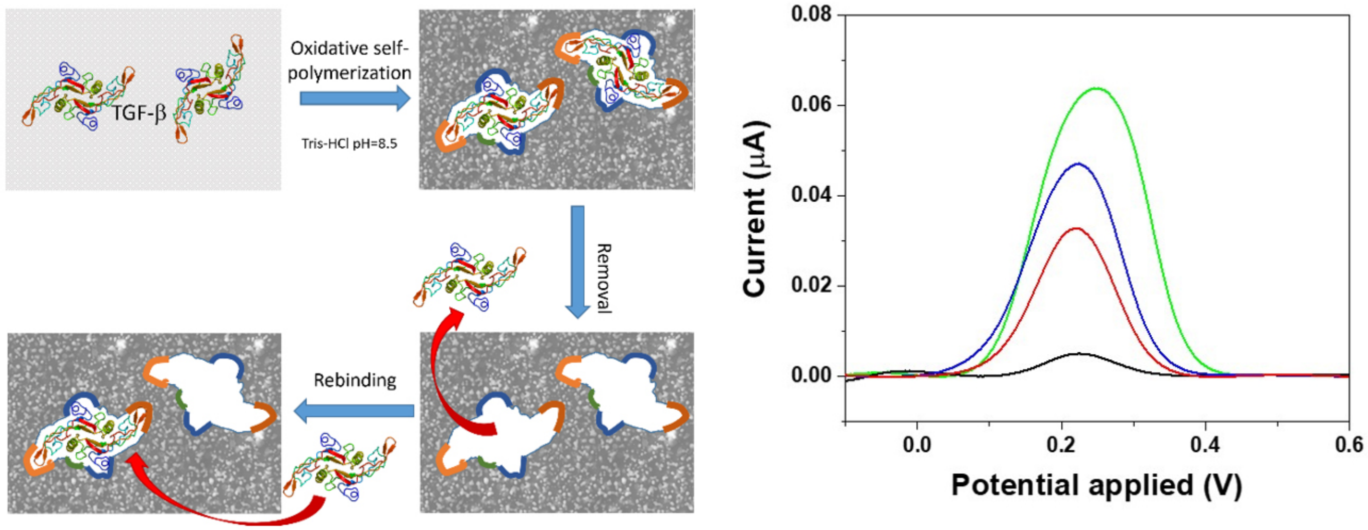

Figure S6: Schematic layout of the adsorption and resorption of TGF-β in the PDA film with corresponding DPV measurements (see text).

the entire process, the performance of MIP at each step (synthesis, template removal, and rebinding) was characterized using the differential pulse voltammetry (DPV) technique in the presence of 10 mM $K_3[Fe(CN)_6]/K_4[Fe(CN)_6]$ in the potential range (-0.2, -0.8) V, at a scan rate of 100 mV/s. All measurements were conducted at room temperature (22 °C). The results are presented in **Figure S6**. Following synthesis, a decrease in peak current (black line) was observed, attributed to the deposition of the polymer film on the electrode surface, obstructing electron transfer. Template removal exhibited an increase in the redox peak (blue line), indicating the presence of empty cavities in the MIP film conducive to the diffusion of the redox probe at the electrode surface. After incubation with TGF-β at 10 ng/mL (red line), a reduction in peak current was observed, suggesting efficient steric hindrance between the target molecule and the biomimetic sites, hindering electron transfer.

MIP morphology. The MIP cladding atop the PhCS was characterized using atomic force microscopy (AFM) in non-contact mode (XE-100 AFM system, Park Systems). The AFM employed a reflectively coated cantilever (Park, resonance frequency 300 kHz). Planar scan frequency was of 0.5 Hz. In **Figure S7**, detailed micrographs covering a $2 \times 2 \mu m^2$ area with a resolution of 512×512 pixels are depicted within

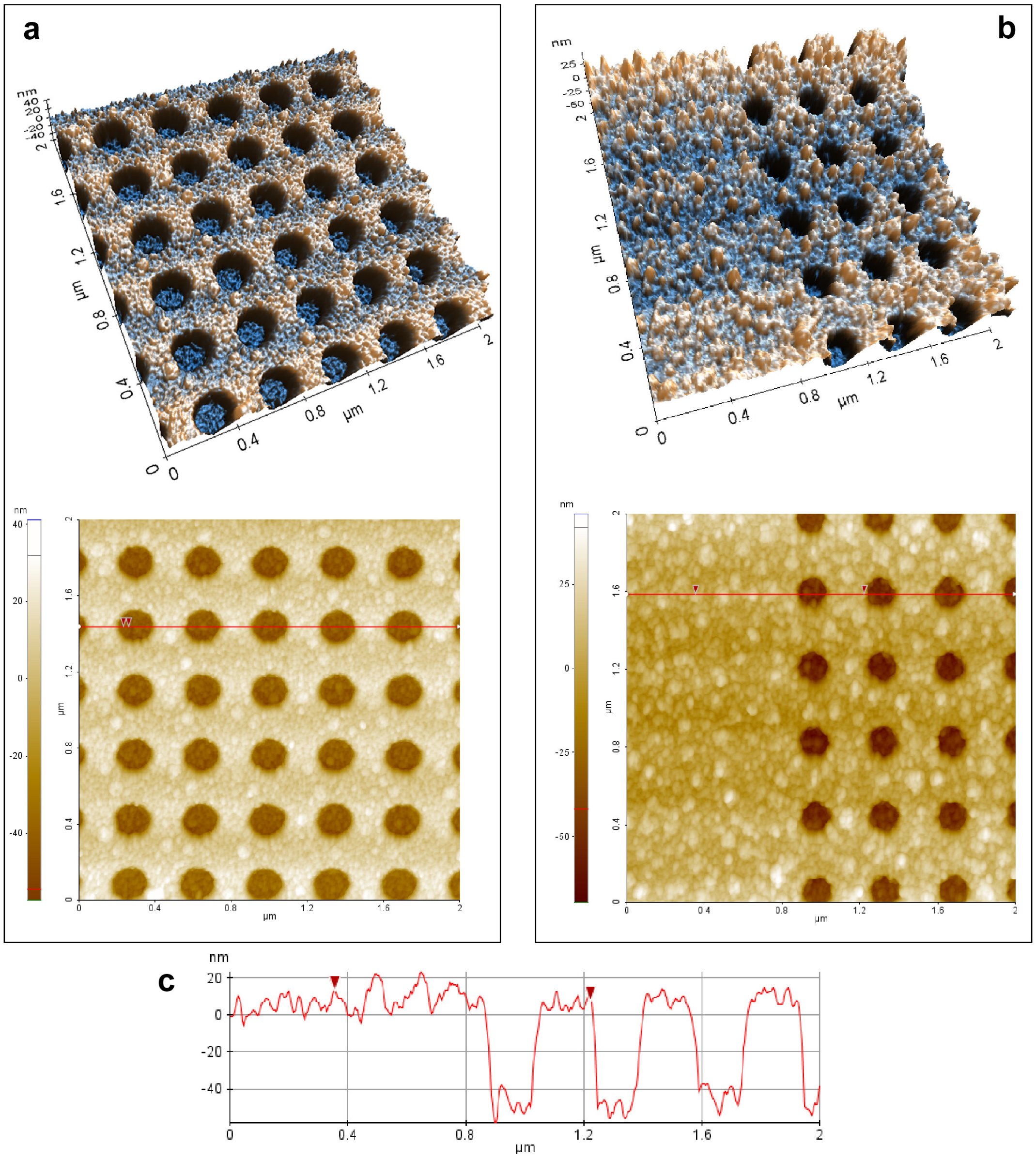

Figure S7: (a) AFM characterization of the MIP cladding film over the PhCS. (b) Detail at the boundary of the PhCS. (c) Cross section topography.

the PhCS and along its boundary. The cross-sectional topography reveals a measured depth of 65 nm, corresponding to an MIP film thickness of approximately 10-25 nm, as determined from trials conducted on a representative PhCS. The imprinted PhCS was finally integrated with a PDMS (polydimethylsiloxane) chamber in order to control the infiltration and the incubation of the analyte. A sketch and an image of the final device and the basic characterization setup are reported in **Figure S8**.

Dissociation constant determination: Figure S9a shows the fit of the adapted Langmuir relation used to estimate the binding affinity. The data of resonance peak shifts $\Delta\lambda$ are the average of 5 measurements. The fit results are $K = 8.4 \pm 2.6$ pM^{-1}, with maximum resonance shift $\Delta\lambda_{\text{max}} = A = 5 \pm 0.4$ nm. The estimated dissociation constant $K_D = 1/K = 125 \pm 45$ fM points out an excellent binding affinity. To validate this result, an alternative approach was also used.
To investigate the binding performance of the TGF-β imprinted sensor, the equilibrium dissociation constant K_D was calculated also using Scatchard plot analysis of binding data. In the Scatchard analysis, the experimental binding is plotted as B/F versus B, where B and F are the bonds and the free concentra-

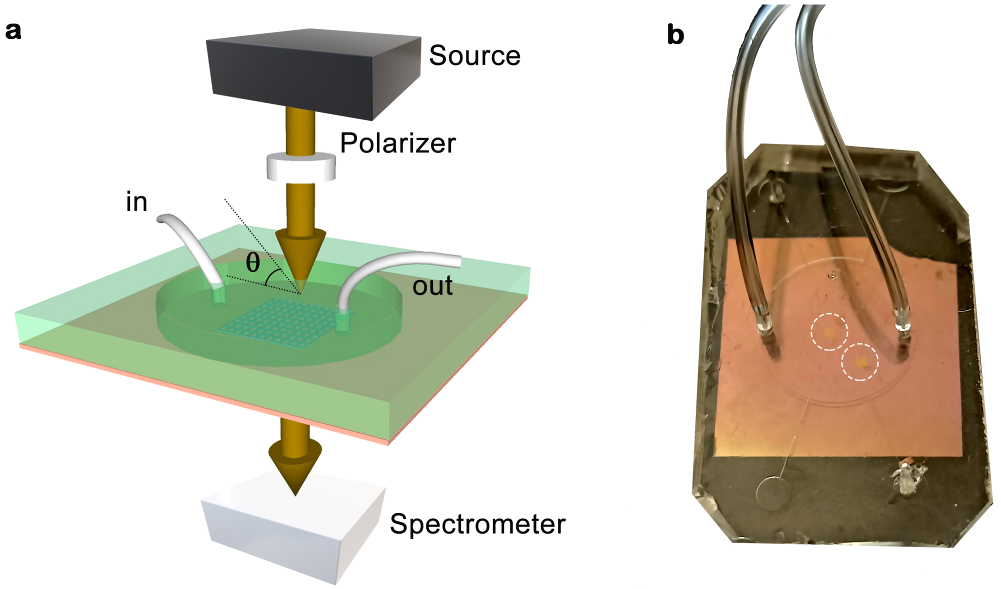

Figure S8: (a) Schematic layout of the PhCS integrated in the microfluidic chamber and basic characterization setup. θ is the incident angle. (b) Image of the final device, dotted circles indicate the PhCS areas.

tions, respectively. Scatchard equation can be written as follows:

$$\frac{B}{F} = \frac{B_{\max}}{K_D} - \frac{B}{K_D}$$

where $B_{\max}$ is the maximum number of binding sites. In the current case, assuming that the bond molecules concentration B is directly proportional to the peak shift $\Delta\lambda$, and that the free concentration F is almost the same as the original concentration C, it is possible to plot the experimental binding isotherm as $\frac{\Delta\lambda}{C}$ versus $\Delta\lambda$ (**Figure S9b**). Assuming that $B = K \times \Delta\lambda$, $B_{\max} = K \times \Delta\lambda_{\max}$, and $F = C$, the equation becomes:

$$\frac{\Delta\lambda}{C} = \frac{\Delta\lambda_{\max}}{K_D} - \frac{\Delta\lambda}{K_D}$$

which was further changed to

$$\frac{1}{C} = \frac{\Delta\lambda_{\max}}{K_D} \times \frac{1}{\Delta\lambda} - \frac{1}{K_D}.$$

The plot of $\frac{1}{C}$ versus $\frac{1}{\Delta\lambda}$ provides a straight line with intercept $-\frac{1}{K_D}$. The estimation of K_D, in this case, is 80 fM, in good agreement with the previous one of 125 ± 45 fM. We retain the larger one as a more conservative estimation.

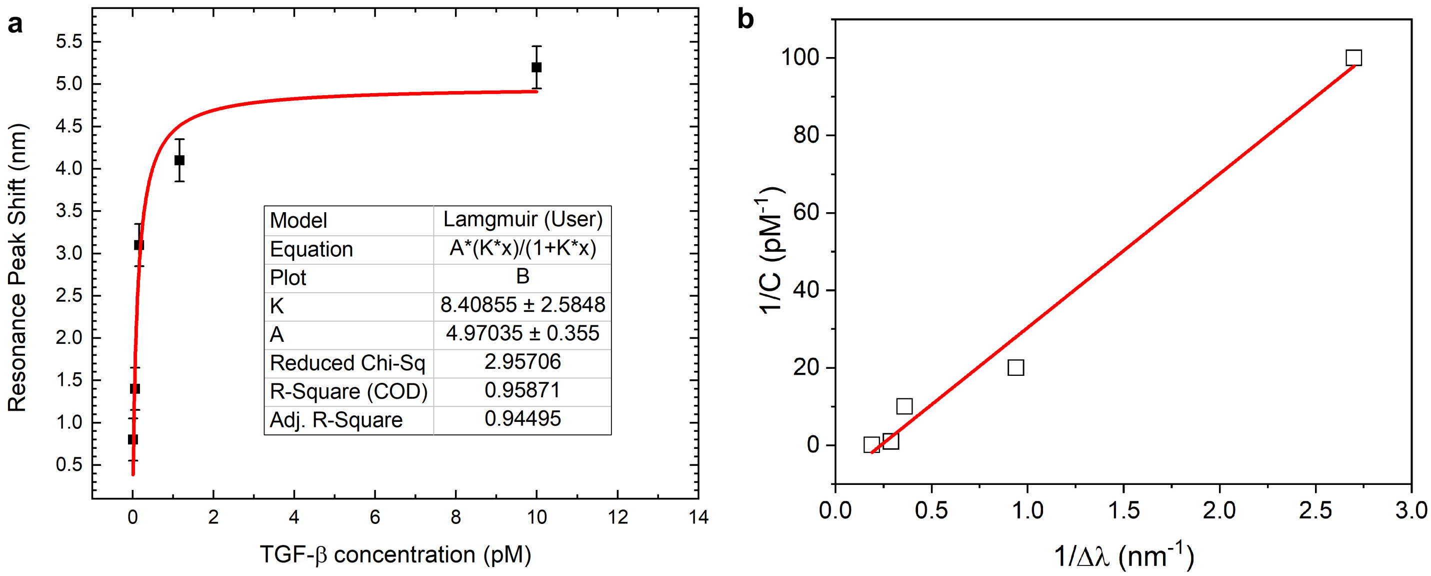

Figure S9: (a) Adapted Langmuir with x = [TGF-β] in units of pM, $y = \Delta\lambda$, K adapted Langmuir constant in units of pM^{-1}. (b) Scatchard plot determined for MIP-BIC sensor, alternatively providing an estimation of $K_D = 1/K$.

Evolution of the dispersion bands with the PhCS thickness

Figure 1b in the main paper showcases the simulations of the actual experimental structure, consisting of a photonic crystal slab (PhCS) of patterned silicon nitride deposited over a quartz substrate. The degeneracy providing a Dirac cone at t = 70 nm arises from the convergence of three modes, each possessing distinct TE-like/TM-like vector characteristics. In a 3D slab structure, each mode can be distinguished by its predominant TE-like or TM-like character. The spatial configurations assumed by the optical field decrease with the thickness, which favors their coalescence. Indeed, with decreasing slab thickness t, the modes coalesce into nearly equal dispersion bands moving towards $\boldsymbol{\Gamma M}$ direction (incidence azimuthal angle $\phi = 45°$) for both TE- and TM-like characters. Their vector character is no longer distinguishable. They retain a more distinctive character only along $\boldsymbol{\Gamma X}$ ($\phi = 0°$) (**Figure S10**).

As the slab thickness increases (**Figure S11** and **Figure S12**), their spatial distributions begin to diverge noticeably. Notably, only along $\boldsymbol{\Gamma M}$ direction, the modes keep having closer dispersion curves, whereas along $\boldsymbol{\Gamma X}$ the bands considerably split apart, breaking the degeneracy in $\boldsymbol{\Gamma}$, owing to the perturbation of their spatial distribution with increasing thickness t. This in turn affects their spectral position. A sizeable splitting is visible in the merge plot of TE and TM modes at $\phi = 0°$ in **Figure S12**. When this occurs, the first TE mode λ_1 of smaller wavelength combines with the middle TM mode 2 (at λ_2) in $\boldsymbol{\Gamma}$ and is not converted into a BIC. The other two modes instead, spectrally separating from each other, must have zero derivatives in $\boldsymbol{\Gamma}$, which also provides them the attribute of BICs because they possess in-plane inversion symmetry. The bottom line is that the peculiar shape of the dispersion cross-section along $\boldsymbol{\Gamma X}$ is always connected with continuity to the whole dispersion surfaces in the three-dimensional momentum space, and both vector characters must be taken into account since they are clearly hybrid modes away from the symmetry points, which affects their properties.

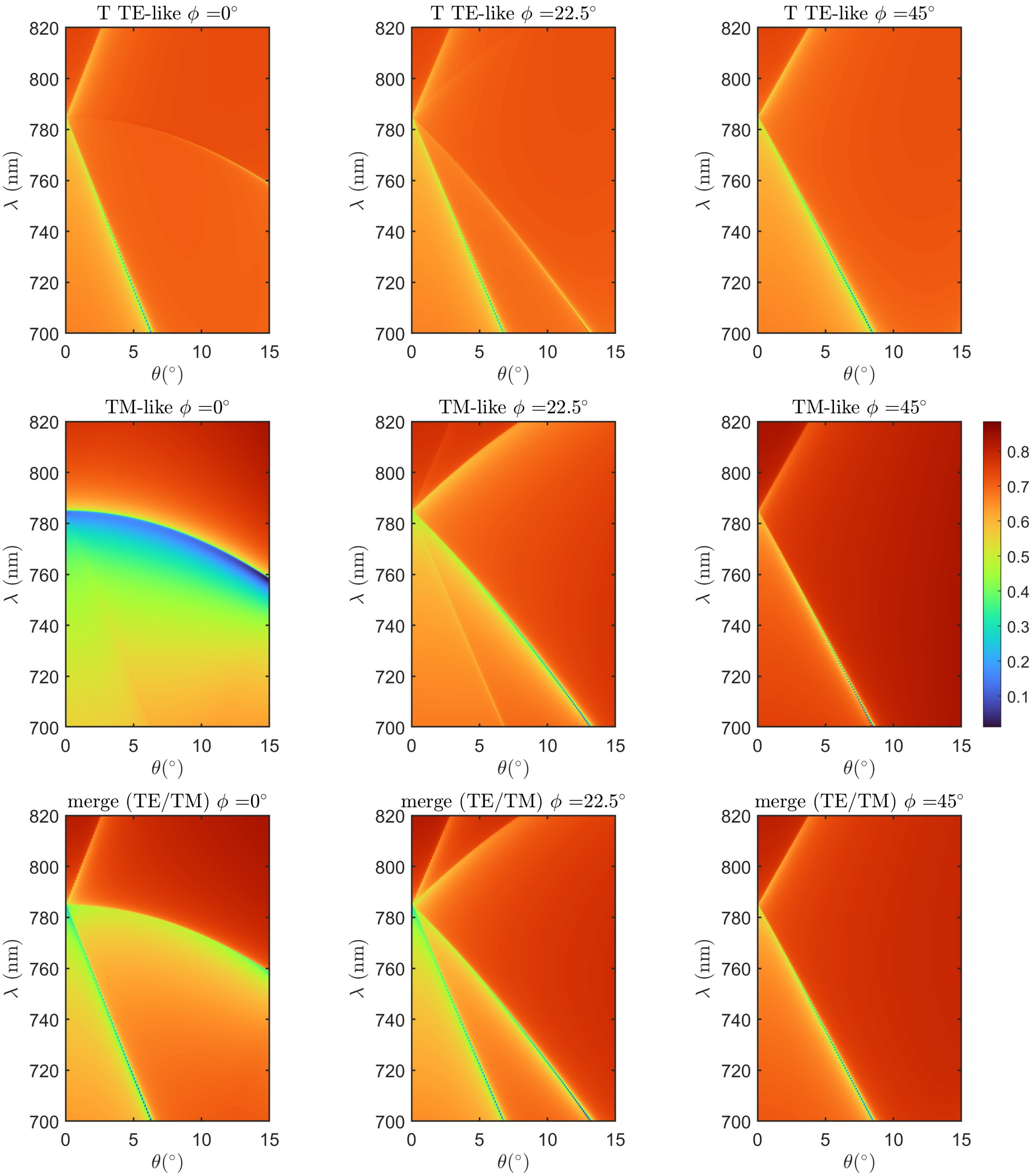

Figure S10: TE, TM and merged TE/TM transmittance band diagrams of the PhCS, from $\mathbf{\Gamma X}$ ($\phi = 0°$) to $\mathbf{\Gamma M}$ ($\phi = 45°$), case $t = 70$ nm.

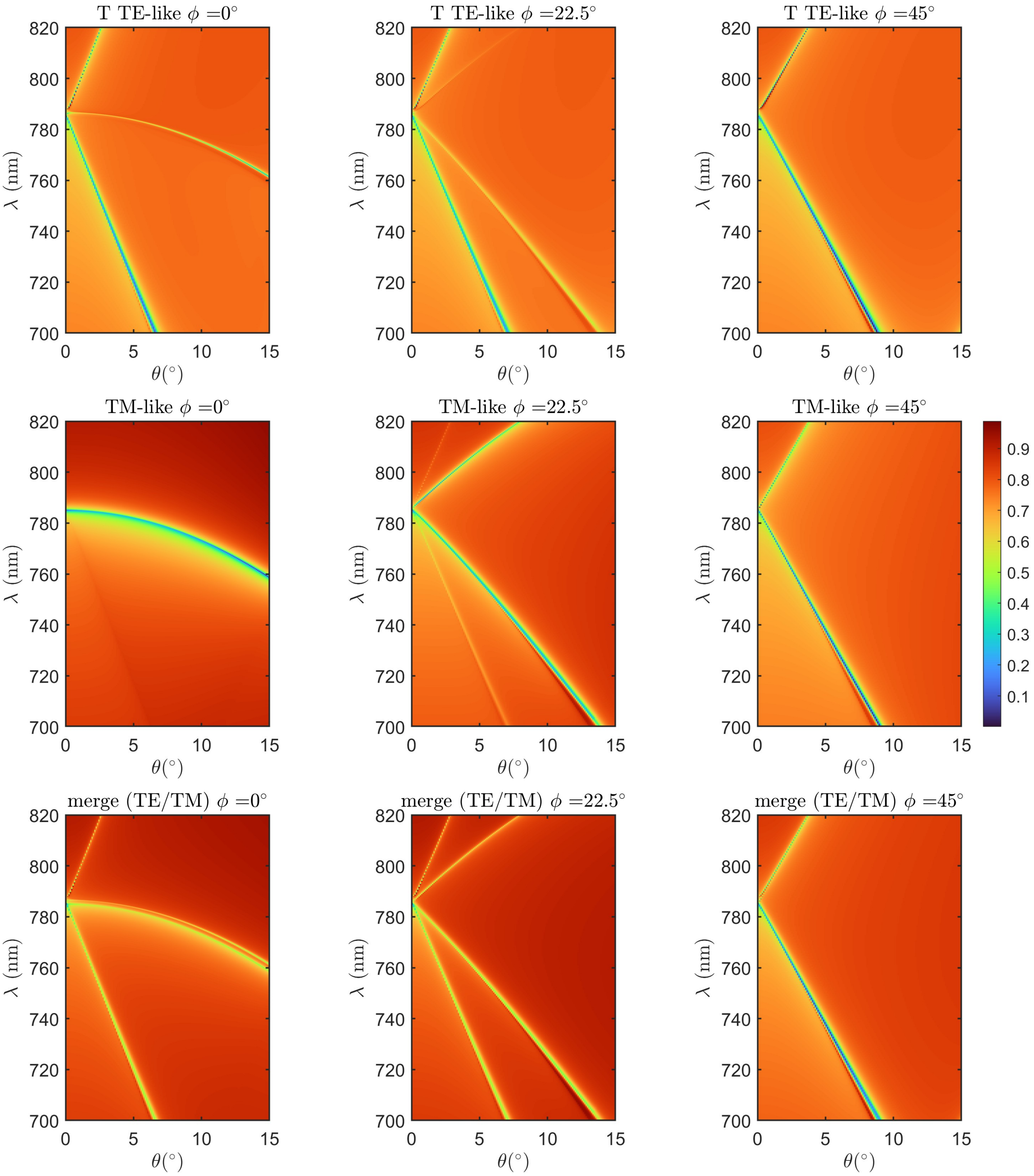

Figure S11: TE, TM and merged TE/TM transmittance band diagrams of the PhCS, from $\mathbf{\Gamma X}$ ($\phi = 0°$) to $\mathbf{\Gamma M}$ ($\phi = 45°$), case $t = 80$ nm.

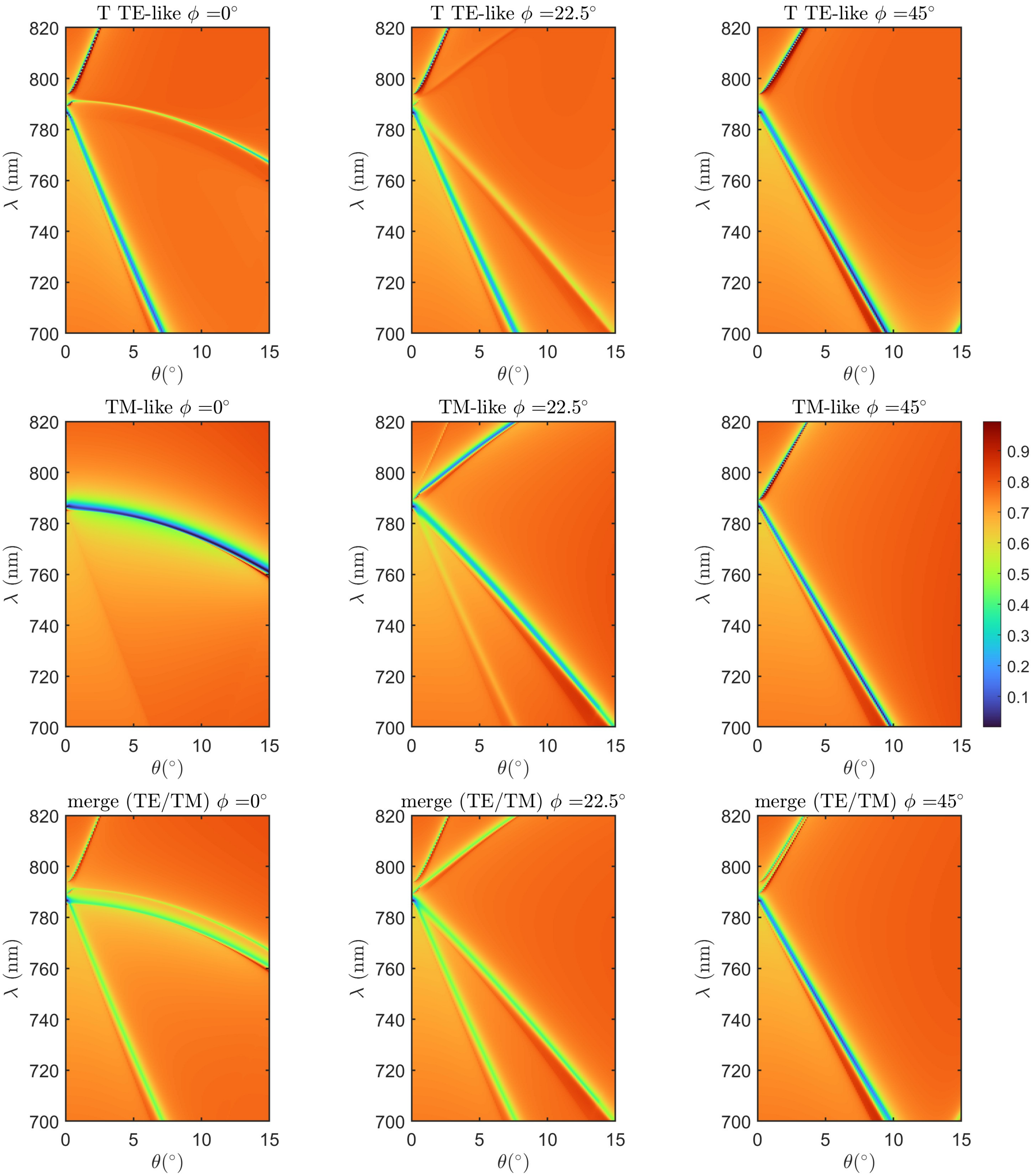

Figure S12: TE, TM and merged TE/TM transmittance band diagrams of the PhCS, from $\mathbf{\Gamma X}$ ($\phi = 0°$) to $\mathbf{\Gamma M}$ ($\phi = 45°$), case $t = 90$ nm.
